# A link between ATP and SUR2A: A novel mechanism explaining cardioprotection at high altitude^☆^

**DOI:** 10.1016/j.ijcard.2015.04.069

**Published:** 2015-06-15

**Authors:** Khaja Shameem Mohammed Abdul, Sofija Jovanović, Qingyou Du, Andriy Sukhodub, Aleksandar Jovanović

**Affiliations:** Medical Research Institute, Division of Cardiovascular and Diabetic Medicine, Ninewells Hospital & Medical School, University of Dundee, Dundee DD1 9SY, UK

**Keywords:** SUR2A, Hypoxia, High Altitude, Cardioprotection

High altitude is defined as living at an altitude of 2400 m or more above sea level. It is well established that prevalence of ischaemic heart disease is decreased in people living at high altitude and that the mortality from this disease is lower in this cohort. Several potential explanations have been offered for the benefit of altitude on the frequency and outcome of ischemic heart disease. It has been shown that hypoxia induces molecular changes that have cardioprotective effect; this was ascribed largely to the actions of HIF-1, a transcription modulator, and up regulation of several genes including erythropoietin, vascular endothelial growth factor, NO synthase, and heme oxygenase-1 [1]. In our previous studies we have shown that sub-hypoxic drop of oxygen as well as mild hypoxia up-regulate SUR2A and confer cardioprotection [2,3]. SUR2A is an “atypical” ABC protein that binds to inward rectifier Kir6.2 to form cardiac sarcolemmal ATP-sensitive K^+^ channels. In vivo, K_ATP_ channels exist as a multiprotein complex that, besides pore-forming Kir6.1/Kir6.2 and regulatory SUR2A subunits, also contain a string of glycolytic and ATP-producing enzymes including creatine kinase, GAPDH and M-LDH. It has been shown that the changes in levels of SUR2A alone have a profound effect on hart development and myocardial susceptibility to different types of metabolic stresses including hypoxia, ischemia, ischemia–reperfusion and stimulation with β-adrenergic agonists [4,5]. Depending on the degree of oxygen decrease signaling pathway regulating SUR2A seems to be different. In a case of sub-hypoxic drop of oxygen, the activation of extracellular signal-regulated kinases (ERK) mediates up-regulation of SUR2A [2]. On the other hand, ERK is not activated by mild hypoxia where, instead, protein kinase B (Akt) and lactate dehydrogenase (LDH) are activated to regulate SUR2A [3]. Whether high altitude-hypoxia would regulate SUR2A levels and what the underlying mechanism would be is yet unknown.

Normobaric 13% oxygen corresponds to oxygen tension at ~ 3600 m above sea level. 24 h-long general exposure of mice to 13% oxygen decreased PO_2_ without affecting PCO_2_ and hematocrit in the blood. In the myocardial tissue, hypoxia did not affect level of lactate dehydrogenase (LDH), but it increased lactate as well as NAD^+^. NADH in the myocardium was decreased while total NAD was not affected. Thus, exposure to 13% oxygen induced all expected signs of hypoxia in the blood and the heart.

It has been shown that mild hypoxia increase levels of SUR2A and confer cardioprotection [3]. Here, 13% oxygen in vivo also increased SUR2A in the myocardium (Fig. 1). Under physiological levels of oxygen, fatty acids serve as the major fuel substrate providing 60–80% of the heart energy requirements, while the rest is derived from glucose and lactate. During chronic hypoxia glucose uptake is enhanced and there is increased contribution of glucose/lactate in ATP production [6]. In vivo, it has been shown that severe hypoxia induces significant drop in myocardial ATP, while mild hypoxia does not affect ATP levels at all [7]. Here, we have found that systemic exposure to 13% oxygen significantly increased myocardial level of ATP (Fig. 1). Most of ATP produced in the heart is catalyzed by CK [8]. Reduction in CK activity significantly reduces myocardial levels of ATP and it induces heart failure in both humans and experimental animals [8]. On the other hand, increased level of CK is associated with increased levels of ATP [9]. Here we have measured levels of CK and found that exposure to 13% hypoxia significantly increased intracellular levels of CK (Fig. 1). Up-regulation of CK by chronic hypoxia has been recently reported [10] and our findings are in agreement with this report. An increased level of CK by hypoxia explains increased ATP in the heart we found. It has been shown in previous studies that sub-hypoxic drop of oxygen activates extracellular regulated kinases 1/2 (ERK1/2) and that mild hypoxia activates protein kinase B (Akt) to up-regulate SUR2A [2,3]. 13% oxygen did not have any effect on ERK1/2 and Akt (data not shown), demonstrating that these kinases did not mediate observed increase in SUR2A. Akt is known to up-regulate LDH that, in turn, directly regulate expression of SUR2A [3]. In the present study, 13% oxygen did not significantly affect LDH levels which further confirms that up-regulation of SUR2A was not associated with signaling pathway activated by 18% oxygen.

ATP is a defining ligand of K_ATP_ channels and it is well established that this nucleotide regulates the channel activity [4]. SUR2A is a regulatory unit of K_ATP_ channels and a rate-limiting factor in formation of these channels [4]. Gating of K_ATP_ channels by ATP has been viewed as a link between membrane excitability and intracellular metabolism [4]. Here, we have examined whether a treatment known to alter intracellular ATP would have any effect on SUR2A levels and cellular resistance to stress. Embryonic rat heart H9c2 cells were treated by 2-deoxyglucose and this treatment 1) decreased intracellular ATP, 2) decreased SUR2A levels and 3) decreased cellular resistance to stress (Fig. 2). These results were compatible with the notion that intracellular ATP regulates SUR2A levels. To further test this hypothesis, we have applied DFNB, an inhibitor of CK. When DFNB was used in a concentration that did not affect intracellular ATP levels, SUR2A levels were also not affected (Fig. 2). On the other hand, when DFNB was applied in a concentration that decreased ATP, it also decreased SUR2A levels (Fig. 2). These findings strongly suggest that intracellular ATP regulate levels of SUR2A in the heart. Does ATP directly regulate SUR2A levels or whether it regulates ATP-depending factors regulating SUR2A has to be addressed in the future.

In conclusion, this study has shown that hypoxia in vivo regulates level of cardioprotective SUR2A by regulating intracellular ATP. This is a previously unrecognized link between intracellular metabolism and cardioprotection.

## Conflict of interest

The authors report no relationships that could be construed as a conflict of interest.

## Figures and Tables

**Fig. 1:**
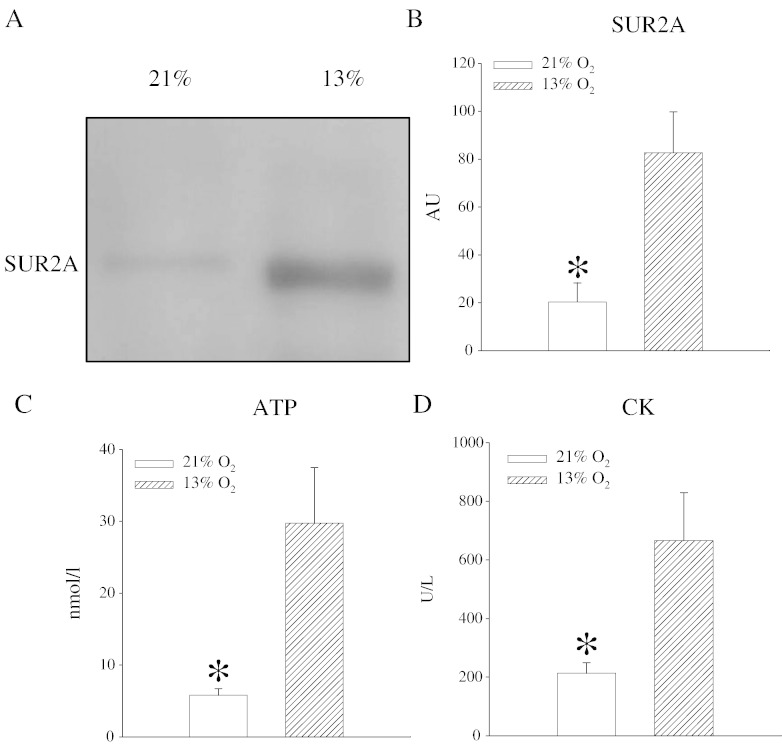
Systemic exposure to 13% oxygen for 24 h increases SUR2A, ATP and CK in the myocardium. Original Western blots for SUR2A under labeled conditions (A) and corresponding graph (B) as well as bar graphs depicting ATP (C) and CK levels (D) in the myocardial tissue under the same conditions. Each bar is a mean ± SEM (n = 3–5). *P < 0.05. AU = arbitrary units. Western blots and methods used to measure ATP and CK are described in details in refs. [2] and [3].

**Fig. 2:**
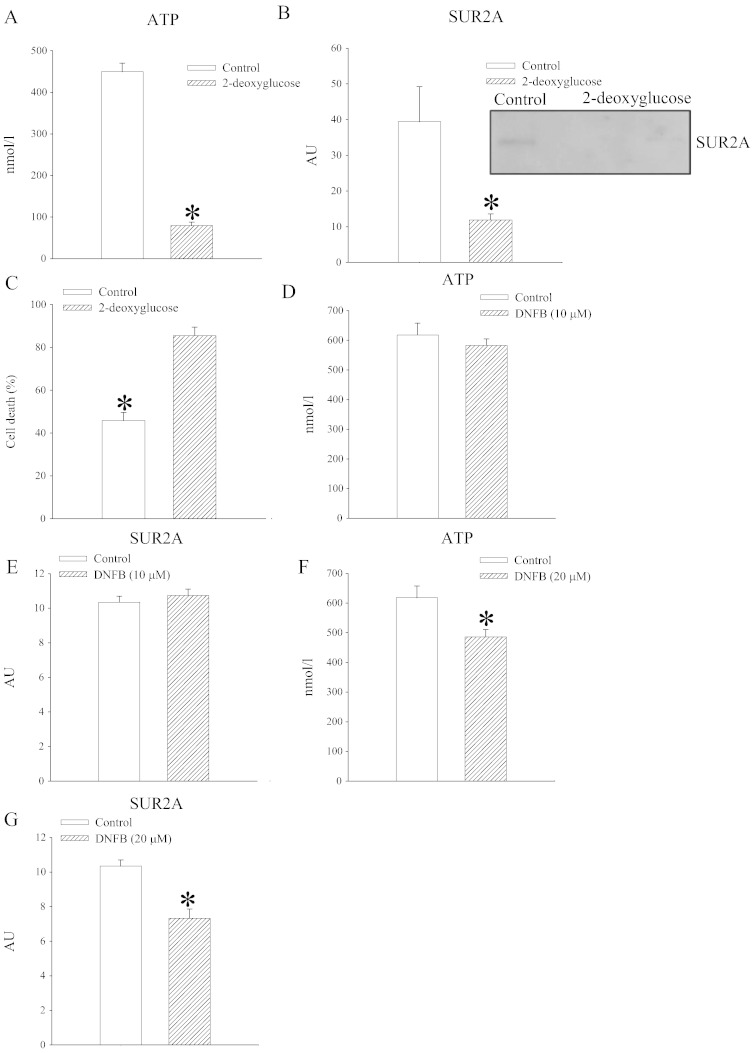
Conditions decreasing intracellular ATP are also associated with decrease in SUR2A and cellular resistance to metabolic stress. Bar graphs depicting ATP (A, D, F) and SUR2A (B, E, G) levels in H9c2 cells treated for 24 h with 50 mM 2-deoxyglucose (A, B, C) or DNFB at concentration of 10 μM (D, E) or 20 μM (F, G) as well as cell survival/death of these cells in response to chemical hypoxia induced by 4 mM DNP (C). Inset in B represent original Western blots for SUR2A under labeled conditions. Each bar is a mean ± SEM (n = 3–5). *P < 0.05. AU = arbitrary units. Western blots and methods used to measure ATP and CK are described in details in refs. [2] and [3].
